# Weed Detection in Perennial Ryegrass With Deep Learning Convolutional Neural Network

**DOI:** 10.3389/fpls.2019.01422

**Published:** 2019-10-31

**Authors:** Jialin Yu, Arnold W. Schumann, Zhe Cao, Shaun M. Sharpe, Nathan S. Boyd

**Affiliations:** ^1^Co-Innovation Center for Sustainable Forestry in Southern China, Nanjing Forestry University, Nanjing, China; ^2^Citrus Research and Education Center, University of Florida, Lake Alfred, FL, United States; ^3^Crop Development Centre/Department of Plant Sciences, University of Saskatchewan, Saskatoon, SK, Canada; ^4^Gulf Coast Research and Education Center, University of Florida, Wimauma, FL, United States

**Keywords:** artificial intelligence, machine vision, machine learning, precision herbicide application, weed control

## Abstract

Precision herbicide application can substantially reduce herbicide input and weed control cost in turfgrass management systems. Intelligent spot-spraying system predominantly relies on machine vision-based detectors for autonomous weed control. In this work, several deep convolutional neural networks (DCNN) were constructed for detection of dandelion (*Taraxacum officinale* Web.), ground ivy (*Glechoma hederacea* L.), and spotted spurge (*Euphorbia maculata* L.) growing in perennial ryegrass. When the networks were trained using a dataset containing a total of 15,486 negative (images contained perennial ryegrass with no target weeds) and 17,600 positive images (images contained target weeds), VGGNet achieved high F_1_ scores (≥0.9278), with high recall values (≥0.9952) for detection of *E. maculata, G. hederacea*, and *T. officinale* growing in perennial ryegrass. The F_1_ scores of AlexNet ranged from 0.8437 to 0.9418 and were generally lower than VGGNet at detecting *E. maculata*, *G. hederacea*, and *T. officinale*. GoogleNet is not an effective DCNN at detecting these weed species mainly due to the low precision values. DetectNet is an effective DCNN and achieved high F_1_ scores (≥0.9843) in the testing datasets for detection of *T. officinale* growing in perennial ryegrass. Moreover, VGGNet had the highest Matthews correlation coefficient (MCC) values, while GoogleNet had the lowest MCC values. Overall, the approach of training DCNN, particularly VGGNet and DetectNet, presents a clear path toward developing a machine vision-based decision system in smart sprayers for precision weed control in perennial ryegrass.

## Introduction

Turfgrasses are the predominant vegetation cover in urban landscapes including golf courses, institutional and residential lawns, parks, roadsides, and sport fields (Milesi et al., 2005). Weed control is one of the most challenging issues for turfgrass management. Weeds compete with turfgrass for nutrient, sunlight, and water, and disrupt turfgrass aesthetics and functionality. Cultural practices, such as appropriate fertilization, irrigation, and mowing, can reduce weed infestation (Busey, 2003), but herbicides often provide the most effective weed control (McElroy and Martins, 2013). Conventional herbicide-based weed control relies on broadcast application, spraying weed patches and pure turfgrass stands indiscriminately. Manual spot-spraying is time-consuming and expensive but practiced commonly to reduce herbicide input and weed control cost.

Deep learning is a category of machine learning that allows a computer algorithm to learn and understand a dataset in terms of a hierarchy of concepts (Deng et al., 2009; LeCun et al., 2015). In recent years, deep learning has emerged as an effective application in various scientific domains, including computer vision (LeCun et al., 2015; Kendall and Gal, 2017; Gu et al., 2018), natural language processing (Collobert and Weston, 2008; Collobert et al., 2011), and speech recognition (Hinton et al., 2012; LeCun et al., 2015). Deep learning has proven to be a promising method in computer-assisted drug discovery and design (Gawehn et al., 2016), sentiment analysis and question answering (Ye et al., 2009; Bordes et al., 2014), predicting sequence specificities of DNA- and RNA-binding proteins (Alipanahi et al., 2015), and performing automatic brain tumor detection (Havaei et al., 2017).

Deep convolutional neural networks (DCNN) have an extraordinary ability to extract complex features from images (LeCun et al., 2015; Schmidhuber, 2015; Ghosal et al., 2018). It has been widely employed as a powerful tool to classify images and detect objects (LeCun et al., 2015). In the 2012 ImageNet competition, a DCNN effectively classified a 1000 class dataset containing approximately a million high resolution images (Krizhevsky et al., 2012). In recent years, a growing number of companies such as Apple, Intel, Nvidia, and Tencent utilize DCNN-based machine vision in facial recognition (Song et al., 2014; Parkhi et al., 2015), self-driving cars (LeCun et al., 2015; Jordan and Mitchell, 2015), real-time smart phone vision applications (Ronao and Cho, 2016), and target identification for robot grasping (Wang et al., 2016).

In agriculture, DCNN reliably detected various ecological crop stresses (Ghosal et al., 2018). For example, Mohanty et al. (2016) presented a DCNN that identified 14 crop species and 26 diseases with an overall accuracy of >99%. Moreover, DCNN-based machine vision can identify plants. For example, Grinblat et al. (2016) presented a DCNN that can reliably identify three legume species using plant vein morphological patterns, while dos Santos Ferreira et al. (2017) presented a DCNN that identified various broadleaf and grassy weeds in relation to soybean (*Glycine max* L. Merr.) and soil, with an overall accuracy of >99%. Recently, Teimouri et al. (2018) presented a method of automatically determining 18 weed species and their growth stages. Accurate image classification and object detection along with the fast image processing are of paramount importance for real-time weed detection and precision herbicide application (Fennimore et al., 2016). Previous researchers noted that training a DCNN takes several hours with a high-performance graphic processor unit (GPU), while the image classification itself is fast (<1 second per image) (Mohanty et al., 2016; Chen et al., 2017; Sharpe et al. 2019; Yu et al., 2019a).

In this work, four DCNN architectures, including i) AlexNet (Krizhevsky et al., 2012), ii) DetectNet (Tao et al., 2016), iii) GoogleNet (Szegedy et al., 2015), and iv) VGG-16 (VGGNet) (Simonyan and Zisserman, 2014) were explored for weed detection in a cool-season turfgrass perennial ryegrass (*Lolium perenne* L.). DetectNet is an object detection DCNN (Tao et al., 2016), while AlexNet, GoogleNet, and VGGNet are image classification DCNNs (Krizhevsky et al., 2012; Simonyan and Zisserman, 2014; Szegedy et al., 2015). In previous works, Yu et al. (2019b) documented that DetectNet was highly effective in detecting annual bluegrass (*Poa annua* L.) and various broadleaf weeds in dormant bermudagrass [*Cynodon dactylon* (L.) Pers.], while VGGNet was highly effective in detecting dollar weed (*Hydrocotyle* spp.), old world diamond-flower (*Hedyotis cormybosa* L. Lam), and Florida pusley (*Richardia scabra* L.) in actively growing bermudagrass. Similarly, Yu et al. (2019a) reported that DetectNet reliably detected cutleaf evening-primrose (*Oenothera laciniata* Hill) in bahiagrass (*Paspalum notatum* Flugge) with overall accuracy >0.99 and recall value of 1.00. However, weed detection in cool-season turfgrass systems with these DCNNs has never been previously reported.

Dandelion (*Taraxacum officinale* Web.), ground ivy (*Glechoma hederacea* L.), and spotted spurge (*Euphorbia maculata* L.) are distributed throughout the continental United States (USDA-NRCS 2018). These weed species are commonly found in various cool-season turfgrasses. Herbicides, such as 2,4-D, dicamba, MCPP, triclopyr, and sulfentrazone are broadcast-applied in cool-season turfgrasses for POST control of various broadleaf weeds (McElroy and Martins, 2013; Reed et al., 2013; Johnston et al., 2016; Yu and McCullough, 2016a; Yu and McCullough, 2016b). Precision herbicide application using machine-vision based sprayers will substantially reduce herbicide input and weed control costs. The objective of this research was to examine the feasibility of using DCNN for detection of broadleaf weeds in perennial ryegrass.

## Materials and Methods

### Image Acquisition

Images of *E. maculata*, *G. hederacea*, and *T. officinale* growing in perennial ryegrass, acquired at multiple golf courses and institutional lawns in Indianapolis, Indiana, United States (39.76 °N, 86.15 ° W), were used in the training datasets. Images of *E. maculata, G. hederacea*, and *T. officinale*, acquired at multiple institutional lawns and golf courses in Carmel, Indiana, United States (39.97° N, 86.11° W), were used in the testing dataset 1 (TD 1). Images of *E. maculata* and *G. hederacea*, acquired at multiple institutional lawns, roadsides, and parks in West Lafayette, Indiana, United States (40.42° N, 86.90° W) were used in the testing dataset 2 (TD 2). Images of *T. officinale* taken at multiple institutional and residential lawns at Saskatoon, Saskatchewan, Canada (52.13° N, 106.67° W) were included in TD 2. The images acquired in Indiana and Saskatchewan were taken using a Sony^®^ Cyber-Shot (SONY Corporation, Minato, Tokyo, Japan) and a Canon^®^ EOS Rebel T6 digital camera (Ohta-ku, Canon Inc., Tokyo, Japan), respectively, at a resolution of 1920 × 1080 pixels. The camera heights were adjusted to a ground-sampling distance of 0.05 cm pixel^-1^ during image acquisition. The images were acquired during the daytime from 9:00 AM to 5:00 PM and under various sunlight conditions including clear, partly cloudy, or cloudy days. The training and testing images were acquired at multiple times between August and September 2018.

### Training and Testing

For training and testing image classification DCNN, images were cropped into 426 × 240 pixels using Irfanview (Version 5.50, Irfan Skijan, Jaice, Bosnia). The images containing a single weed species were selected for training and testing image classification DCNN. The neural networks were trained using the training dataset containing either a single weed species (single-species neural network) or multiple weed species (multiple-species neural network). For training single-species neural networks, the *E. maculata* training dataset contained 6,180 negative images (images containing perennial ryegrass without target weeds) and 6,500 positive images (images containing perennial ryegrass infested with target weeds); the *G. hederacea* training dataset contained 4,470 negative and 4,600 positive images; and the *T. officinale* training dataset contained 4,836 negative and 6,500 positive images. The above training datasets were used to train DCNN for detecting a single weed species growing in perennial ryegrass. For each weed species, a total of 630 negative and 630 positive images were used for validation dataset (VD), TD 1, and TD 2.

The multiple-species neural networks were trained because we were interested to evaluate the feasibility of using a single image classification DCNN to detect multiple weed species growing in perennial ryegrass. We trained AlexNet, GoogLeNet, and VGGNet using two training datasets (A and B). Training dataset A was a balanced dataset that contained 19,500 negative and 19,500 positive images (6,500 images per weed species). Training dataset B was an unbalanced dataset containing a total of 15,486 negative and 17,600 positive images (6,500 images for *E. maculata*; 4,600 images for *G. hederacea*; and 6,500 images for *T. officinale*). A total of 900 negative and 900 positive images (300 images for each weed species) were used for VD. The TD 1 and TD 2 for the multiple-species neural networks contained 630 negative and 630 positive images.

When training object detection DCNN, images were resized to 1280 × 720 pixels (720 p) using Irfanview. A total of 810 images containing *T. officinale* while growing in perennial ryegrass were used for training DetectNet. Training images were imported into custom software compiled using Lazarus (http://www.lazarus-ide.org/). Bounding boxes were drawn on imported images to identify objects. Program output generated corresponding text files used for DetectNet training. A total of 100 images containing *T. officinale* while growing in perennial ryegrass were used in the VD, TD 1 or TD 2. For detection of *T. officinale*, the images of VD, TD 1, and TD 2 contained a total of 630, 446, and 157 individual weeds, respectively.

Neural network training and testing were performed in the NVIDIA Deep Learning GPU Training System (DIGITS) (version 6.0.0, NVIDIA Corporation, Santa Clara, CA) using the Convolutional Architecture for Fast Feature Embedding (Caffe) (Jia et al., 2014). Networks were pre-trained using the ImageNet database (Deng et al., 2009) and KITTI dataset (Geiger et al., 2013). The following hyper-parameters were standardized to compare the results of all DCNN.

- Base learning rate: 0.03
- Batch accumulation: 5
- Batch size: 2
- Gamma: 0.95
- Learning rate policy: Exponential decay
- Solver type: AdaDelta
- Training epochs: 30

The results of validation and testing for all DCNN were arranged in binary confusion matrixes, including true positive (*tp*), true negative (*tn*), false positive (*fp*), and false negative (*fn*) (Sokolova and Lapalme, 2009). In this context, *tp* represents the images containing target weeds that are correctly identified; *tn* represents the images containing turfgrasses without target weeds that are correctly identified; *fp* represents the images without target weeds that are incorrectly identified as target weeds; and *fn* represents the images containing target weeds incorrectly not identified as turfgrasses. We computed the precision (Equation 1), recall (Equation 2), F_1_ score (Equation 3), and Matthews correlation coefficient (MCC) (Equation 4) for each DCNN. The precision, recall, and F_1_ score values are unitless indices of predictive ability and ranged from 0 to 1. The higher the value is, the better is the predictive ability of the network. The high precision indicates that the neural network achieved high successful rate of detection for the turfgrass area where weeds do not occur, while the high recall indicates that the neural network realized high successful rate of detection for the target weeds. MCC is a correlation coefficient between the observed and predicted binary classifications. MCC ranges from -1 to +1. An MCC value of -1 represents the total discrepancy between observation and prediction, 0 indicates no better than a random prediction, and +1 represents a prefect prediction (Matthews, 1975).

Precision measures the ability of the neural network to accurately identify targets, which was calculated using the following equation (Sokolova and Lapalme, 2009):

(1) $$\text{Precision}=\frac{tp}{tp+fp}$$

Recall measures the ability of the neural network to detect its target, which was calculated using the following equation (Sokolova and Lapalme, 2009; Hoiem et al., 2012):

(2) $$\text{Recall}=\frac{tp}{tp+fn}$$

F_1_ score is a harmonic means of the precision and recall (Sokolova and Lapalme, 2009) calculated by the following equation:

3 $$F1\text{ Score}=\frac{2\times \text{Precision}\times \text{Recall}}{\text{Precision}+\text{Recall}}$$

MCC measures the quality of binary classifications, particularly the correlation between the actual class labels and predictions, which was determined by the following equation (Matthews, 1975).

4 $$\text{MCC}=\frac{tp\times tn-fp\times fn}{\sqrt{\left(tp+fp)\times (tp+fn)\times (tn+fp)\times (tn+fn\right)}}$$

## Results and Discussion

When single-species neural networks were trained for detection of *E. maculata*, AlexNet and VGGNet exhibited higher precision, recall, F_1_ score, and MCC values compared to GoogleNet (**Table 1**). AlexNet achieved slightly higher F_1_ score than VGGNet, primarily due to higher precision. For detection of *G. hederacea*, AlexNet and VGGNet exhibited excellent performances and achieved high F_1_ score values (≥0.9906) for VD, TD 1, and TD 2. AlexNet and VGGNet exhibited high F_1_ scores (≥0.9115) and recall values (≥0.9952) at detecting *T. officinale* for VD and TD 1, but the recall values decreased to 0.6032 and 0.9603, respectively for TD 2. Among the neural networks, GoogleNet consistently exhibited the lowest MCC values, indicating the poor predictions for the actual class labels. The poor performance of GoogleNet was primarily due to the low precision (≤0.7464). DetectNet achieved high F_1_ scores (≥0.9933) and reliably detected *T. officinale* for VD, TD 1, and TD 2.

**Table 1:** Weed detection training results while growing in perennial ryegrass using artificial neural networks.^a^

Weed species	Single-species neural network	VD^b^				TD 1				TD 2			
		Precision	Recall	F_1_ score	MCC	Precision	Recall	F_1_ score	MCC	Precision	Recall	F_1_ score	MCC
*Euphorbia maculata*.	AlexNet	0.8787	1.0000	0.9354	0.8702	0.7702	1.0000	0.8702	0.7351	0.7816	1.0000	0.8774	0.7505
	GoogleNet	0.5147	0.9984	0.6793	0.1671	0.5032	1.0000	0.6695	0.0799	0.5032	1.0000	0.6695	0.0799
	VGGNet	0.8630	1.0000	0.9265	0.8521	0.7491	1.0000	0.8566	0.7058	0.6796	1.0000	0.8092	0.5994
*Glechoma hederacea*	AlexNet	0.9937	0.9984	0.9960	0.9921	0.9937	0.9952	0.9944	0.9889	0.9905	0.9968	0.9937	0.9873
	GoogleNet	0.7429	1.0000	0.8525	0.6970	0.6113	0.9984	0.7583	0.4697	0.7464	1.0000	0.8548	0.7021
	VGGNet	0.9968	1.0000	0.9984	0.9968	0.9813	1.0000	0.9906	0.9811	0.9906	1.0000	0.9953	0.9905
*Taraxacum officinale*	AlexNet	0.9472	0.9968	0.9714	0.9426	0.8396	0.9968	0.9115	0.8209	0.9694	0.6032	0.7436	0.6309
	GoogleNet	0.5394	1.0000	0.7008	0.2807	0.5168	1.0000	0.6814	0.1834	0.6859	0.9984	0.8132	0.6080
	VGGNet	0.9488	1.0000	0.9737	0.9474	0.8546	0.9984	0.9209	0.8406	1.0000	0.9603	0.9798	0.9611
*Taraxacum officinale*	DetectNet	0.9968	1.0000	0.9984	0.8152	0.9955	0.9911	0.9933	0.8147	0.9875	1.0000	0.9937	0.8399

a Single-species neural network was trained using the training dataset containing a single weed species. The training dataset of E. maculata contained 6,180 negative and 6,500 positive images; the training dataset of G. hederacea contained 4,470 negative and 4,600 positive images; and the training dataset of T. officinale contained 4,836 negative and 6,500 positive images. For object detection DCNN, the training dataset contained a total of 810 images.

b For image classification DCNN, VD, TD 1, or TD 2 contained 630 negative and 630 positive images. For object detection DCNN, VD, TD 1, or TD 2 contained a total of 100 images. DCNN, deep convolutional neural work; VD, validation dataset; TD 1, testing dataset 1; TD 2, testing dataset 2; MCC, Matthews correlation coefficient.

The capability of multiple-species neural networks to simultaneously detect multiple weed species was evaluated using the VD (contained three weed species per dataset) (**Table 2**). VGGNet exhibited the highest MCC values (≥0.9640), while GoogleNet exhibited the lowest MCC values (≤0.2718). AlexNet and VGGNet achieved high F_1_ scores (≥0.9395), with high recall values (≥0.9822) at detecting these weeds. AlexNet and VGGNet exhibited higher precision but lower recall values when trained using the training dataset A than the training dataset B. AlexNet and VGGNet out-performed GoogleNet. GoogleNet was not effective in detecting selected weeds, with the F_1_ score values of 0.6894 and 0.7006 when trained using the training dataset A and B, respectively.

**Table 2:** Validation results of multiple-species neural networks for detection of weeds while growing in perennial ryegrass.^a^

Training dataset	Multiple-species neural network	Precision	Recall	F_1_ score	MCC
A^b^	AlexNet	0.9824	0.9900	0.9862	0.9723
	GoogleNet	0.5288	0.9900	0.6894	0.2204
	VGGNet	0.9944	0.9822	0.9883	0.9767
B^c^	AlexNet	0.8858	1.0000	0.9395	0.8784
	GoogleNet	0.5427	0.9878	0.7006	0.2718
	VGGNet	0.9646	1.0000	0.9820	0.9640

a Multiple-species neural network was trained using the training dataset containing multiple weed species. The validation dataset contained a total of 900 negative and 900 positive images (300 images for each weed species).

b Training dataset A contained a total of 19,500 negative and 19,500 positive (6,500 images for each weed species) images.

c Training dataset B contained a total of 15,486 negative and 17,600 positive images (6,500 images for Euphorbia maculata; 4,600 images for Glechoma hederacea; and 6,500 images for Taraxacum officinale). MCC, Matthews correlation coefficient.

The capability of multiple-species neural networks to detect these weed species was further evaluated using TD 1 and TD 2 (contained only a single weed species per dataset) (**Table 3**). When the AlexNet was trained using the training dataset A, it performed similarly to when trained using the training dataset B. AlexNet demonstrated high recall (≥0.9619) but relatively low precision (≤0.7412) for detection of *E. maculata* and *T. officinale* in the TD 1 and *T. officinale* in the TD 2, which reduced the F_1_ score values. The MCC value of GoogleNet was the lowest, while the MCC value of VGGNet was the highest among the multiple-species neural networks for both training datasets.

**Table 3:** Testing results of multiple-species neural networks for detection of weeds while growing in perennial ryegrass.^a^

Training dataset	Multiple-species neural network	Weed species	TD 1				TD 2			
			Precision	Recall	F_1_ score	MCC	Precision	Recall	F_1_ score	MCC
A^b^	AlexNet	*Euphorbia maculata*	0.7412	1.0000	0.8514	0.6945	0.8666	1.0000	0.9285	0.8562
		*Glechoma hederacea*	0.7305	0.9984	0.8437	0.6773	0.8355	1.0000	0.9104	0.8192
		*Taraxacum officinale*	0.8990	0.9889	0.9418	0.8822	0.8359	0.9619	0.8945	0.7820
	GoogleNet	*Euphorbia maculata*	0.5036	1.0000	0.6699	0.6699	0.5040	1.0000	0.6702	0.0894
		*Glechoma hederacea*	0.4874	0.9222	0.6378	-0.1054	0.5025	0.9508	0.6575	0.0211
		*Taraxacum officinale*	0.5024	0.9952	0.6677	0.0490	0.5479	0.9984	0.7075	0.3068
	VGGNet	*Euphorbia maculata*	0.9781	0.9937	0.9858	0.9716	0.9890	0.9968	0.9929	0.9857
		*Glechoma hederacea*	0.9844	1.0000	0.9921	0.9843	0.9692	1.0000	0.9844	0.9687
		*Taraxacum officinale*	0.9825	0.9794	0.9809	0.9619	1.0000	0.8048	0.8918	0.8206
B^c^	AlexNet	*Euphorbia maculata*	0.7560	0.9984	0.8605	0.7139	0.8279	1.0000	0.9058	0.8098
		*Glechoma hederacea*	0.8364	0.9984	0.9103	0.8187	0.8225	1.0000	0.9026	0.8031
		*Taraxacum officinale*	0.7962	0.9984	0.8859	0.7680	0.7714	0.9857	0.8655	0.7221
	GoogleNet	*Euphorbia maculata*	0.5348	1.0000	0.6969	0.2638	0.5237	0.9651	0.6790	0.1622
		*Glechoma hederacea*	0.5194	1.0000	0.6837	0.1968	0.5488	1.0000	0.7087	0.3123
		*Taraxacum officinale*	0.5674	0.9889	0.7211	0.3509	0.4703	0.7794	0.5866	-0.1306
	VGGNet	*Euphorbia maculata*	0.8796	0.9968	0.9345	0.8681	0.9445	1.0000	0.9715	0.9429
		*Glechoma hederacea*	0.8974	1.0000	0.9459	0.8916	0.8886	1.0000	0.9410	0.8816
		*Taraxacum officinale*	0.8654	1.0000	0.9278	0.8549	0.9984	0.9952	0.9968	0.9937

a Multiple-species neural network was trained using the training dataset containing multiple weed species. TD 1 or TD 2 contained 630 negative and 630 positive images.

b Training dataset A contained a total of 19,500 negative and 19,500 positive images (6,500 images for each weed species).

c Training dataset B contained a total of 15,486 negative and 17,600 positive images (6,500 images for E. maculata, 4,600 images for G. hederacea, and 6,500 images for T. officinale). TD 1, testing dataset 1; TD 2, testing dataset 2; MCC, Matthews correlation coefficient.

When the neural networks were trained for detecting multiple weed species, VGGNet exhibited excellent performances in detecting *E. maculata* and *G. hederacea* and achieved high F_1_ scores (≥0.9345) and recall values (≥0.9968) in the TD 1 and TD 2. However, for detection of *T. officinale*, VGGNet trained using the training dataset B exhibited considerably higher recall and F_1_ score values than when trained using the training dataset A. GoogLeNet was ineffective at detecting these weed species, primarily due to the low precision values.

Both image classification and object detection DCNN can be used in the machine vision sub-system of smart sprayers but each approach has pros and cons. Compared to the object detection DCNN, the training of the image classification DCNN takes less time because it does not involve the drawing of bounding boxes. The herbicide application of DetectNet-based smart sprayers can target individual weeds as objects using the narrow spray pattern nozzles, while this is less feasible with the image classification DCNN.

Except for *T. officinale*, selected DCNN produced consistent results for weed detection, even across different geographical regions. When single-species neural networks were trained, AlexNet and VGGNet exhibited excellent precision and recall at detecting *T. officinale* in the TD 1, but the recall values decreased in the TD 2. The reduction in recall value suggests that the network is more likely to misclassify target weeds as turfgrass. This is undesirable in field applications as weeds would be missed, inadequate herbicide applied, and thus result in poor weed control. The cause is unknown, but we hypothesized that it was likely due to the variations in the morphological characteristics of *T. officinale* between the training and testing images. Fortunately, this problem has been overcome by training the multiple-species neural networks, particularly the VGGNet.

**Figure 1:**
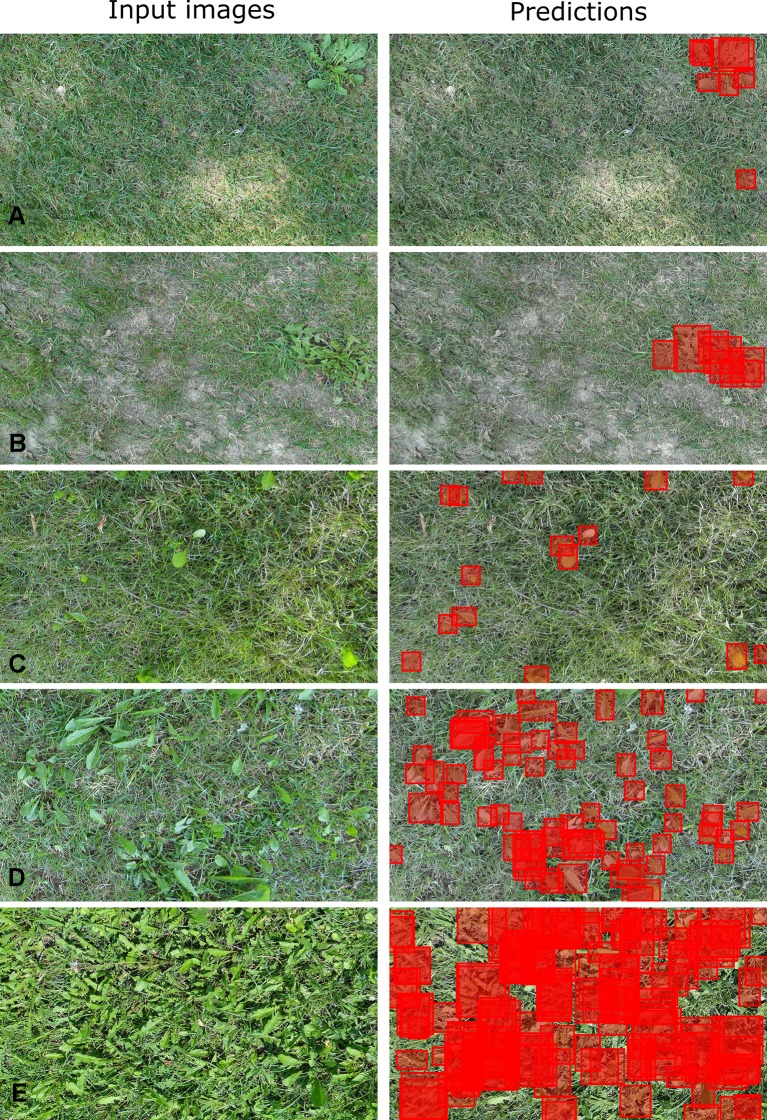
DetectNet-generated bounding boxes (predictions) generated on the testing images (input images) of *Taraxacum officinale* while growing in perennial ryegrass. **(A)** The DetectNet detected *T. officinale* at mature and seedling growth stages, respectively. **(B)** The DetectNet incorrectly detected a *Digitaria ischaemum* as *T. officinale*. **(C**–**E)** The DetectNet detected *T. officinale* at different growth stages and weed densities.

DetectNet exhibited excellent performances at detecting *T. officinale* across different growth stages and densities (**Figures 1A–E**). Several images used for model testing in TD 1 had high weed densities. The bounding boxes generated by DetectNet failed to cover every single leaf of the target weeds when the testing images containing high weed densities (**Figure 1E**), which reduced the recall values. However, this is unlikely to be an issue in field applications because the great majority of weeds per image are detected. The few undetected leaves would likely fall into the spray zone if the herbicides are delivered using flat fan nozzles. In addition, several testing images in TD 2 contained smooth crabgrass [*Digitaria ischaemum* (Schreb.) Muhl]. In a few cases, DetectNet incorrectly detected the smooth crabgrass as the *T. officinale* (**Figure 1B**), which reduced precision. Increasing the number of training images containing crabgrass growing with *T. officinale* may remove this effect and increase the precision and overall accuracy. In previous research, DetectNet trained to detect Carolina geranium (*Geranium carolinianum* L.) in plastic-mulched strawberry crops was successfully desensitized to black medic (*Medicago lupulina* L.) leaves in a similar circumstance (Sharpe et al., 2018).

**Figure 2:**
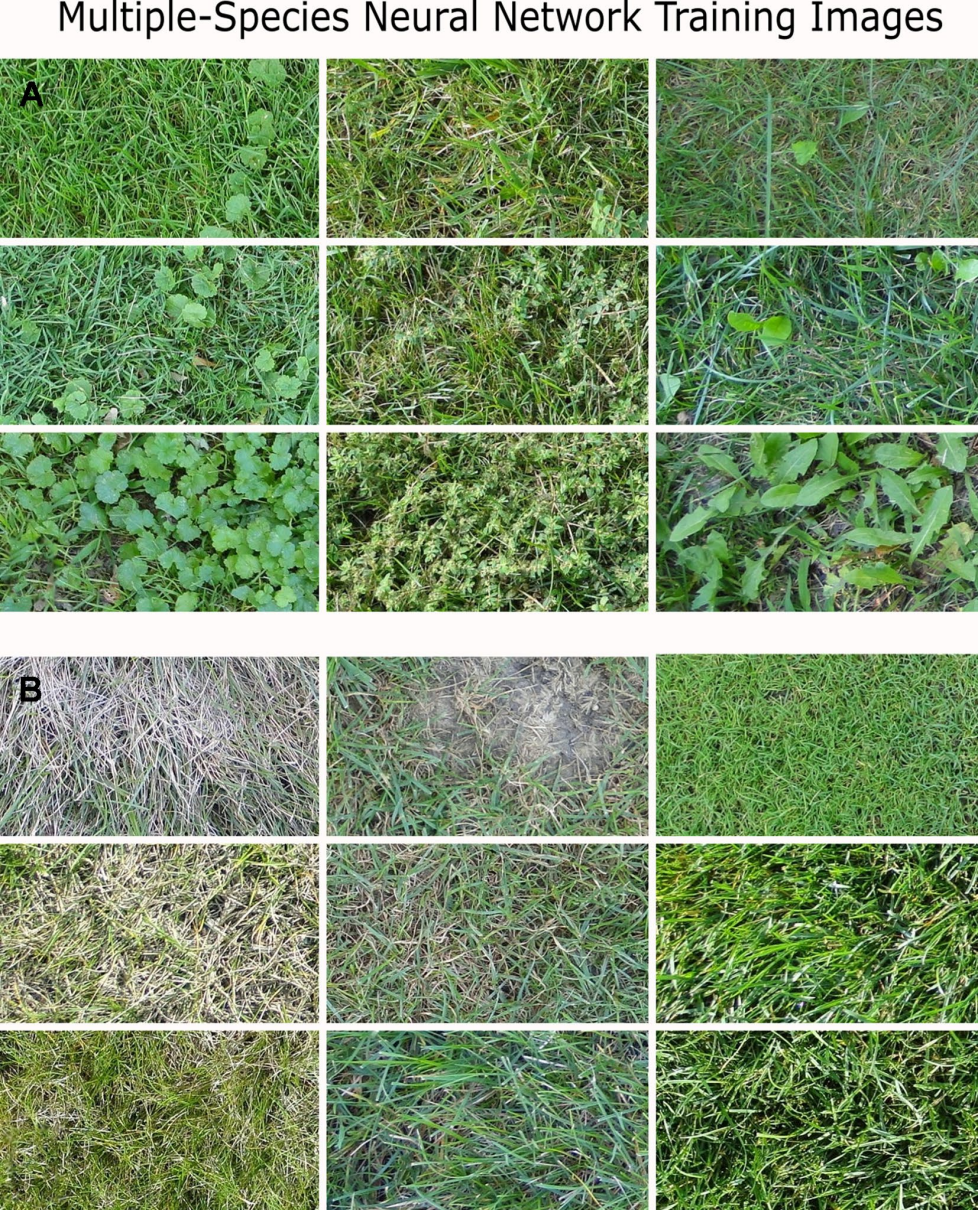
Images for training the multiple-species neural networks. **(A)** *Euphorbia maculata, Glechoma hederacea*, and *Taraxacum officinale* at various weed densities. **(B)** Perennial ryegrass at different turfgrass management regimes, mowing heights, and surface conditions.

In the present study, we evaluated the effectiveness of using a single DCNN to detect multiple weed species growing in perennial ryegrass. Simultaneous detection of multiple weed species is of paramount importance for precision herbicide application because weeds often grow in mixed stands and various postemergence herbicides, such as 2,4-D, carfentrazone, dicamba, and MCPP, are sprayed to provide broad-spectrum control of various broadleaf weeds (McElroy and Martins, 2013; Reed et al., 2013). For training purposes, *E. maculata, G. hederacea*, and *T. officinale* constituted a single category of objects to be discriminated from perennial ryegrass (**Figure 2**). The selected weed species exhibited tremendous differences in plant morphology, while perennial ryegrass was viewed at different turfgrass management regimes, mowing heights, and surface conditions (**Figure 2**). Meanwhile, weeds present in the training and testing images were at different growth stages, which added the extra complexity for the machine learning algorithms. Surprisingly, VGGNet (trained using training dataset B) achieved high F_1_ scores (≥0.92), with high recall values (≥0.99) in the VD, TD 1, and TD 2. These results suggest that the simultaneous detection of multiple weed species with a single VGGNet is effective even when target weeds have distinct morphological structures and weed densities.

AlexNet and VGGNet trained using the training dataset A resulted in higher precision and F_1_ score, but lower recall than the training dataset B in the VD and most testing results in the TD 1 and TD 2. Recall is a critical factor for precision herbicide application. High recall indicates that target weeds are more likely to be correctly identified, whereas low recall indicates that target weeds are more likely to be misidentified, leading to inadequate herbicide application and thus poor weed control. Regardless of the training datasets, GoogLeNet exhibited high recall but unacceptable precision. Low precision is undesirable since resultant networks are more likely to misclassify turfgrasses as target weeds and spraying herbicides where weeds do not occur.

The training dataset A is balanced and contained equal number of negative and positive images, whereas the training dataset B is unbalanced and contained less training images than the training dataset A. Because of the differential performances of multiple-species neural networks, particularly AlexNet and VGGNet, were evident when trained using the training dataset A and B, we hypothesized that (1) the ratios of negative and positive images in the training dataset may influence the performances of neural networks, (2) changing the number of positive images for each weed species in the training dataset may alter the performances for weed detection, and (3) a larger training dataset might be needed to further improve the precision and recall and enhance the overall accuracy. These hypotheses will be tested in further work. It should be noted that all training images were taken in a relatively small geographical area. While the model achieved high classification rates, a more diversified training dataset that represents different weed biotypes collected in different geographical regions is highly desired. Moreover, pixel-wise semantic segmentation was noted to improve the accuracy of object detection with less training images (Milioto and Stachniss, 2018; Milioto et al., 2018), which warrant further evaluation. The expansion of the neural networks to include a wider variety of weed species should be the next immediate step of this research.

## Conclusion and Summary

This research demonstrated the feasibility of using DCNN for weed detection in perennial ryegrass. When the neural networks were trained using training datasets containing a single weed species, AlexNet and VGGNet performed similarly for detection of *E. maculata* and *G. hederacea* growing in perennial ryegrass. AlexNet and VGGNet had reduced the recall values in the TD 2 for detection of *T. officinale*, but this problem was overcome by training the multiple-species neural networks. VGGNet consistently exhibited the highest MCC values in the multiple-species neural networks for both training datasets. VGGNet trained using training dataset A exhibited higher precision and F_1_ score but lower recall compared to the models trained using training dataset B. VGGNet (trained using the training dataset B) achieved high F_1_ score (≥0.9278), with high recall (≥0.9952), indicating that it is highly suitable for the automated detection of *E. maculata*, *G. hederacea*, and *T. officinale* growing in perennial ryegrass. GoogleNet is not an effective DCNN at detecting these weed species primarily due to the unacceptable precision. DetectNet exhibited excellent F_1_ scores (≥0.9843) and recall values (≥0.9911) in the TD 1 and TD 2, and thus is an effective DCNN for detection of *T. officinale* growing in perennial ryegrass. To further improve the accuracy of weed detection, other DCNN architectures, such as Single Shot Detection (SSD; Liu et al., 2016), You Only Look Once (Yolo; Redmon et al., 2016), and residual network (He et al., 2016) may be investigated in the future.

## Data Availability Statement

The datasets generated for this study are available on request to the corresponding author.

## Author Contributions

JY, AS, and NB designed the experiment. JY, ZC, and SS acquired the images. JY trained DCNN models, analyzed the data, and drafted the manuscript.

## Conflict of Interest

The authors declare that the research was conducted in the absence of any commercial or financial relationships that could be construed as a potential conflict of interest.
